# The Effects of *Sargassum horneri* Extract and Fucoidan on Tear Hyposecretion and Ocular Surface Injury in Rats with Dry Eye Diseases

**DOI:** 10.3390/cimb45080415

**Published:** 2023-08-08

**Authors:** Su-Bin Park, Woo Kwon Jung, Hwa Young Yu, Junghyun Kim

**Affiliations:** Department of Oral Pathology, School of Dentistry, Jeonbuk National University, Jeonju 54896, Republic of Korea; tnqls309@gmail.com (S.-B.P.); wkjungjbnu@gmail.com (W.K.J.); naive17jbnu@gmail.com (H.Y.Y.)

**Keywords:** dry eye, fucoidan, *Sargassum horneri*

## Abstract

Hyperosmotic stress caused by tear hyposection is a leading cause of dry eye disease. We investigated the prevention of dry eye disease in corneal epithelial cells and in rats that were induced to develop dry eye disease via unilateral excision of their exorbital lacrimal gland using *Sargassum horneri* extract (AB_SH) and its bioactive component fucoidan. Oral administration of AB_SH (250 mg/kg and 500 mg/kg) and fucoidan (100 mg/kg) was conducted for 7 days. In order to measure tear secretion, phenol red thread tear tests were performed along with corneal irregularity measurements. The apoptotic injury in the cornea and the lacrimal gland was evaluated using TUNEL staining. AB_SH and fucoidan were shown to suppress apoptosis and the expression of apoptosis-related proteins in human corneal epithelial cells under hyperosmotic conditions. Oral administration of AB_SH and fucoidan attenuated tear hyposecretion and corneal irregularity in the lacrimal gland-excised rats. In addition, AB_SH and fucoidan also reduced apoptosis in the cornea and lacrimal gland. This study suggests that *S. horneri* extract and fucoidan can effectively ameliorate dry eye disease by suppressing the apoptosis of ocular tissues.

## 1. Introduction

Keratoconjunctivitis sicca is a dry eye disease characterized by unstable tear film caused by dysfunction of the integrated lacrimal system and ocular surface [1,2]. It causes the ocular surface epithelial cells to undergo accelerated apoptosis as a result of inadequate lubrication due to decreased tear production [3]. The ocular surface and tear film in patients with dry eye disease show an increased density of inflammatory cells. In addition, they show elevated levels of proapoptotic factors and inflammatory cytokines [4,5]. Currently, the detailed cause of keratoconjunctivitis sicca is unknown; however, inflammation and apoptosis within ocular epithelial cells and lacrimal glands may be crucial. Dry eye patients can benefit from artificial tears to replace some of their aqueous tear fluid, but artificial tears only compensate for a portion of the fluid lost on account of dry eyes. The topical application of artificial tears must be repeated several times [6].

An edible brown algae known as *Sargassum horneri* grows along the coasts of South Korea, China, and Japan, and it contains a variety of healthy ingredients, including polyphenols, polysaccharides, and chromenes, that have been demonstrated to provide health benefits [7]. Korea’s traditional medicine has used S. horneri for its healing properties in inflammatory and allergic disorders [8]. *S. horneri* was an essential food and medicine source in Chinese medicine [9]. Fucoidan, fucoxanthin, and alginate, which are constituents of *S. horneri*, have been shown to inhibit the production of pro-inflammatory cytokines [10,11,12]. Using *S. horneri* extract and fucoidan as its active component, we investigated whether they can alleviate the symptoms of dry eye disease, particularly changes in tear secretion, ocular surface damage, and lacrimal gland dysfunction.

## 2. Materials and Methods

### 2.1. Preparation of S. horneri Extract

Standardized *S. horneri* extract (production code name: AB_SH) was provided by the Acebiome Inc. R&D center (Daejeon, Republic of Korea). *S. horneri* was purchased from Ohta Foods Korea Co. Ltd. (Wando, Republic of Korea). For the preparation of AB_SH, about 30 kg of dried *S. horneri* was weighed and extracted in boiling water. The extract solution was filtered and concentrated. To remove arsenic from AB_SH, the concentrated extract solution was then mixed with an arsenic absorption carrier (Ohta Foods Korea Co. Ltd., Wando, Republic of Korea). Then, the mixture was filtered and concentrated. The concentrated extract was spray-dried to give an extract powder of AB_SH. AB_SH was standardized using fucose, a major monomer in fucoidan from *S. horneri*, as a reference compound, via high-performance liquid chromatography (HPLC). AB_SH was hydrolyzed, and a derivatization reaction with 3-methy-1-phenyl-5-pyrazolone (PMP) was performed according to the method described by Lee et al. [13]. This derivatization method was developed for the analysis of carbohydrates via HPLC [14].

### 2.2. Cell Culture and Cell Viability Assay

According to the manufacturer’s instructions, corneal epithelial cells (American Type Culture Collection, Manassas, VA, USA) were maintained in corneal epithelial cell basal medium containing growth supplements. In order to test cell viability, we used a Promega Corporation MTS assay kit (Madison, WI, USA). In 96-well plates, cells (1 × 10^4^ cells/well) were cultured. We treated cells at a density of 2 × 10^5^/well with AB_SH or fucoidan (10~1000 μg/mL). AB_SH and fucoidan were dissolved in the culture medium. After incubation for 24 h, cell viability was determined. Microplate readers (Tecan, Männedorf, Switzerland) were used to measure absorbance at 490 nm for the MTS assay.

### 2.3. Apoptosis under Hyperosmotic Stress Stress

A 500 mOsm hyperosmolar medium was obtained by adding NaCl. Assays were conducted using isotonic (300 mOsm) or hypertonic (500 mOsm) media containing AB_SH (10~100 μg/mL) or fucoidan (1~50 μg/mL) for 24 h. Based on the fucoidan content of AB_SH, we determined our doses of fucoidan. As described above, an MTS assay was used to determine cell viability. Following the manufacturer’s instructions, TUNEL staining (Roche, Mannheim, Germany) was used to detect apoptotic cells.

### 2.4. Western Blot Analysis

We evaluated the expression levels of proteins associated with apoptosis using Western blotting. Several antibodies were used, including rabbit anti-Bax antibody (1:1000, Abcam, Waltham, MA, USA), rabbit anti-Bcl2 antibody (1:1000, Abcam, Waltham, MA, USA), rabbit anti-PARP antibody (1:2000, Abcam, Waltham, MA, USA), and rabbit anti-caspase-3 antibody (1:2000, Abcam, Waltham, MA, USA). As a final step, the protein bands were observed using a Western blotting detection kit (Thermo, Waltham, MA, USA) after being treated with a HRP-conjugated goat anti-rabbit IgG antibody (1:1000, Abcam, Waltham, MA, USA). In order to determine protein expression levels, an image analyzer was used (ATTO, Tokyo, Japan).

### 2.5. Animal Experiment

Male SD rats (6 weeks old) were anesthetized with isoflurane (JW Pharmaceutical, Seoul, Republic of Korea). We performed surgical excision of the left exorbital lacrimal gland in order to induce tear hyposecretion and hyperosmotic stress. Three days following surgery, the animals were randomly divided into four groups of ten each. (1) vehicle-treated dry eye rat (dry eye); (2) 250 mg/kg AB_SH-treated dry eye rats (AB_SH-250); (3) 500 mg/kg AB_SH-treated dry eye rats (AB_SH-500); (4) 100 mg/kg fucoidan-treated dry eye rats (fucoidan-100). AB_SH and fucoidan dissolved in distilled water were administered orally for 7 days. A sham operation was performed on rats in the normal control group (Normal). At necropsy, the right exorbital lacrimal gland and both eyes were isolated and fixed with 10% neutralized buffered formalin. The IACUC approved the protocol for this study (approval no. 2022-05) that used animals in the experiment.

### 2.6. Tear Volume Measurement

Based on the previously reported method [6], the amount of tears was measured. For one minute, phenol red cotton threads (Zone Quick; FCI Ophthalmics, MA, USA) were placed in the outer cantus and measured. There is a millimeter scale for measuring tears.

### 2.7. Corneal Irregularity Scoring

According to the previously reported method [6], corneal irregularity was observed as a result of tear film instability. A stereomicroscope is attached to a circular illuminator, which reflects a circular light onto the cornea. This light is used to determine the shape of the lines reflected on the cornea. The following scoring criteria were used: 0, perfect circle shape; 1, distortion at 1/4 of the circle; 2, distortion at 2/4 of the circle; 3, distortion at 3/4 of the circle; 4, distortion across 4/4 of a circle; and 5, severe distortion that does not recognize circle shape.

### 2.8. TUNEL Staining

Apoptosis was detected using a TUNEL kit (Roche, Mannheim, Germany) in the formalin-fixed and paraffin-embedded cornea and lacrimal gland tissue sections. Under a fluorescence microscope (Olympus, Tokyo, Japan), we quantified how many apoptotic cells were TUNEL-positive.

### 2.9. Statistical Analysis

GraphPad Prism 8.0 software (San Diego, CA, USA) was used for statistical analysis, and a one-way analysis of variance (ANOVA) followed by Tukey’s multiple comparison test was used for group comparison.

## 3. Results

### 3.1. HPLC Analysis of AB_SH

**Figure 1:**
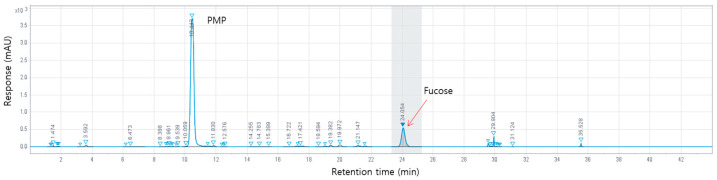
HPLC chromatographs of AB_SH.

The HPLC analysis of AB_SH showed that it contained various compounds (Figure 1). AB_SH had its fucose content measured using HPLC for standardization. The experiment confirmed that the content of fucose was 321.6 ± 0.21 mg/g in AB_SH (Table 1).

### 3.2. AB_SH and Fucoidan Inhibits Hyperosmotic Stress-Induced Corneal Cell Apoptotic Injury

**Figure 2:**
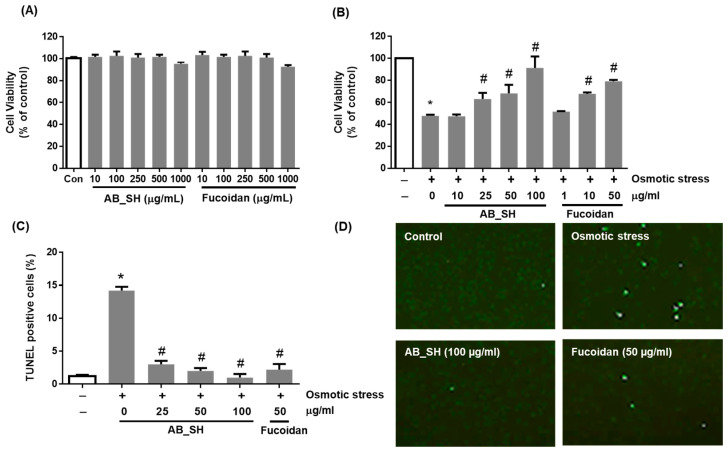
Effects of AB_SH and fucoidan on corneal epithelial cell injury under hyperosmotic conditions. (**A**,**B**) Cell viability was measured using an MTS assay. (**C**,**D**) Apoptosis was detected using TUNEL staining. Data shown are mean ± SE (*n* = 5). The significance of differences among means was evaluated using the one-way ANOVA with a Tukey’s test for multiple comparisons; * *p* < 0.05 vs. normal control group, # *p* < 0.05 vs. dry eye group; * *p* < 0.05 vs. non-treated control group, # *p* < 0.05 vs. osmotic stress-treated group.

To determine the cytotoxicity of AB_SH and fucoidan, we conducted a cell viability assay in human corneal epithelial cells. The results indicated that it was safe up to a concentration of 500 μg/mL (Figure 2A). We investigated the effects of AB_SH and fucoidan on hyperosmotic stress-induced corneal epithelial cell injury. A cell viability assay showed a 2-fold decrease in cell viability under hyperosmotic conditions (*p* < 0.05). However, the treatment with AB_SH or fucoidan resulted in the attenuation of hyperosmotic stress-induced corneal epithelial cell injury in a dose-dependent manner (*p* < 0.05) (Figure 2B). AB_SH and fucoidan were tested for their effects on hyperosmotic stress-induced apoptosis of corneal epithelial cells using TUNEL staining. Under hyperosmotic conditions, the ratio of apoptotic cells was increased more than 10-fold in corneal epithelial cells compared with control cells (*p* < 0.05) (Figure 2C,D). Concentration-dependently, AB_SH and fucoidan reduced the number of apoptotic cells in corneal epithelial cells (*p* < 0.05). Under hyperosmotic conditions, corneal epithelial cells’ viability decreases primarily due to apoptosis, and AB_SH and fucoidan exhibit cytoprotective effects via anti-apoptotic activity.

### 3.3. AB_SH and Fucoidan Regulates the Expression Levels of Pro-Apoptotic and Anti-Apoptotic Proteins

**Figure 3:**
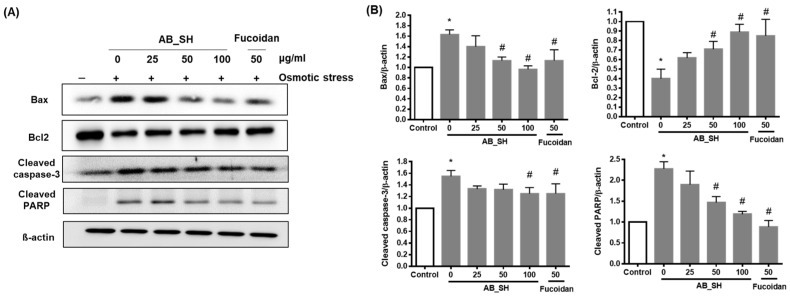
Effects of AB_SH and fucoidan on the protein expression levels of Bax, bcl-2, cleaved caspase-3, and cleaved PARP in corneal epithelial cells under hyperosmotic conditions. (**A**) Representaitive western blot images. (**B**) Quantitative analysis of expression of Bax, bcl-2, cleaved caspase-3, and cleaved PARP. Data shown are mean ± SE (*n* = 5). The significance of differences among means was evaluated using a one-way ANOVA with a Tukey’s test for multiple comparisons; * *p* < 0.05 vs. non-treated control group, # *p* < 0.05 vs. osmotic stress-treated group.

The effects of AB_SH and fucoidan on the expression levels of apoptosis-related proteins were determined in corneal epithelial cells. The levels of Bax, cleaved caspase-3, and cleaved-PARP pro-apoptotic proteins increased, and the level of the anti-apoptotic protein, Bcl-2, decreased under hyperosmotic conditions. However, these expression changes of apoptosis-related proteins were attenuated by the treatment of AB_SH and fucoidan in a dose-dependent manner (Figure 3A,B).

### 3.4. AB_SH and Fucoidan Improves Tear Secretion and Corneal Irregularity in the Lacrimal Gland-Excised Rats

**Figure 4:**
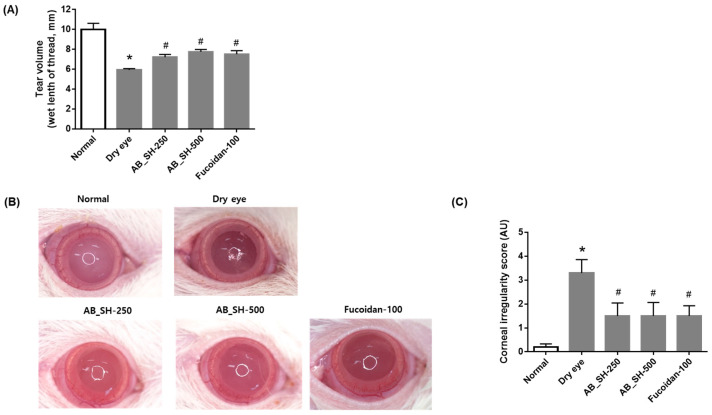
Effects of AB_SH and fucoidan on tear secretion (**A**) and corneal irregularity (**B**,**C**) in unilateral exorbital lacrimal gland-excised rats. Data shown are mean ± SE (n = 10). The significance of differences among means was evaluated using the one-way ANOVA with a Tukey’s test for multiple comparisons; * *p* < 0.05 vs. normal control group, # *p* < 0.05 vs. dry eye group. Normal control rats: Normal, lacrimal gland-excised rats: Dry eye, 250 mg/kg AB_SH-treated rats: AB_SH-250, 500 mg/kg AB_SH-treated rats: AB_SH-500 and 100 mg/kg fucoidan-treated rats: fucoidan-100.

In order to test the effect of AB_SH and fucoidan in a rat model with dry eye disease, the unilateral exorbital lacrimal gland was surgically excised in rats. AB_SH and fucoidan were orally administered for 7 days. As shown in Figure 4A, tear volume was significantly decreased in the lacrimal gland-excised rats (5.93 ± 0.43 mm) compared with that in the normal control rats (10.00 ± 1.94 mm). This hyposecretion of tear was dose-dependently reversed by AB_SH (7.21 ± 0.85 mm and 7.75 ± 0.75 mm, respectively) and fucoidan (7.51 ± 1.14 mm). An evaluation of tear film stability was conducted by measuring corneal irregularity scores (Figure 4B,C). It was evident that the circular shape of the ring illuminator was retained on the ocular surface of normal control rats (0.00 ± 0.00). Significant distortion of the circular shape was observed in the lacrimal gland-excised rats (3.30 ± 1.77). A reduction in circular distortion was achieved using AB_SH (1.50 ± 1.72 and 1.50 ± 1.78, respectively) and fucoidan (1.50 ± 1.35 mm).

### 3.5. AB_SH and Fucoidan Prevents Apoptotic Injury in the Corneal Epithelium and Lacrimal Gland

**Figure 5:**
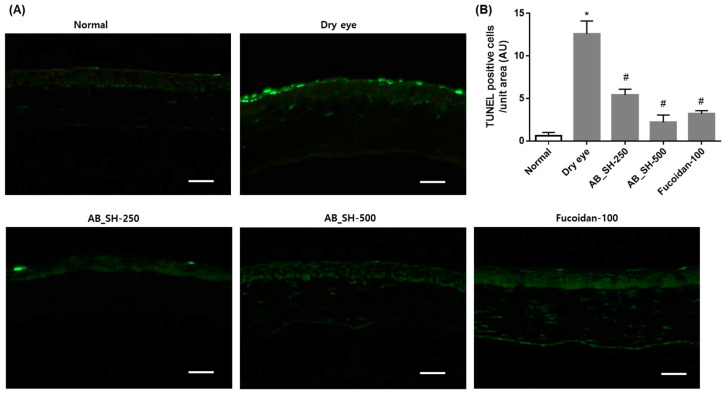

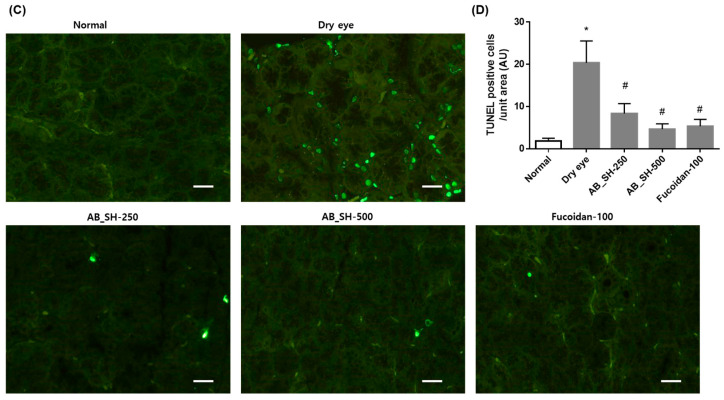
Effect of AB_SH and fucoidan on apoptosis in corneal epithelium (**A**,**B**) and lacrimal gland (**C**,**D**) using TUNEL staining. Scale bar = 100 μm. Data shown are mean ± SE (n = 10). The significance of differences among means was evaluated using a one-way ANOVA with a Tukey’s test for multiple comparisons; * *p* < 0.05 vs. normal control group, # *p* < 0.05 vs. dry eye group. Normal control rats: Normal, lacrimal gland-excised rats: Dry eye, 250 mg/kg AB_SH-treated rats: AB_SH-250, 500 mg/kg AB_SH-treated rats: AB_SH-500 and 100 mg/kg fucoidan-treated rats: fucoidan-100.

Several previous studies have shown that corneal cells and lacrimal cells are damaged by apoptotic injury under dry eye conditions [15,16]. TUNEL staining was performed to confirm whether AB_SH and fucoidan also inhibit the apoptosis of corneal cells and lacrimal cells in rats with dry eye disease. As shown in Figure 5, the apoptotic cells in the corneal epithelium and lacrimal gland were significantly increased in the lacrimal gland-excised rats than those of normal control rats. However, this increase in apoptosis was restored by the administration of AB_SH and fucoidan.

## 4. Discussion

Ocular surface injury can occur as a result of dry eye disease, which is the most commonly occurring eye disorder [17]. Evaporation of moisture from the tear film causes dry eye disease, which is caused by insufficient tear production. Ocular surface damage appears to be associated with increased tear osmolarity, which contributes to the pathogenesis of dry eye syndrome [18,19]. Patients with dry eye disease and animal models suffer from hyperosmolar tears. These tears produce an array of inflammatory cytokines [20,21,22]. Aqueous tears are produced by the lacrimal gland, which protects the conjunctiva and cornea from hyperosmotic stress [23,24]. Lacrimal functional units are composed of lacrimal glands, ocular surfaces, and communicating innervations that control tear production [25]. A damaged or dysfunctional lacrimal functional unit can compromise the homeostasis of the ocular surface [26]. Hyperosmotic stress induced by the hyposecretion of tears leads to the recruitment of immune cells in the lacrimal glands and cornea, which may induce inflammation and inflammatory-mediated injury within these tissues [27].

A number of ophthalmic drops, including anti-inflammatory agents, immunosuppressive agents, and steroids, are used to treat dry eye disease, including artificial tears, lubricants, and steroid eye drops [28]. Although these treatments alleviate symptoms of dry eye disease, they do not address its underlying causes. For this reason, we investigated the protective effects of *S. honeri* extract in order to provide a supplemental therapy for dry eye disease. *S. honeri* has traditionally been used to treat osteoporosis, hyperlipidemia, high blood pressure, allergies, and inflammation [29,30,31]. *S. honeri* extract contains fucoidan, which is anti-inflammatory, antioxidant, and tissue-protective due to its multiple protective properties [32]. Further, we evaluated whether fucoidan is a major bioactive component of AB_SH.

In the present study, AB_SH and fucoidan treatments on corneal cells increased viability and inhibited apoptosis under hyperosmotic conditions. Apoptosis inhibition by AB_SH and fucoidan was confirmed by measuring the levels of pro-apoptotic and anti-apoptotic proteins. When corneal cells are treated with AB_SH, Bax, caspase-3, and PARP are down-regulated. A concentration-dependent increase in anti-apoptotic Bcl2 protein was observed with AB_SH treatment. Fucoidan also showed this anti-apoptotic effect in corneal cells under hyperosmotic conditions. Moreover, the oral administration of AB_SH and fucoidan in the dry eye rats also showed beneficial activity against tear hyposecretion and apoptosis of corneal epithelium and lacrimal gland. Therefore, our results reveal the pharmacological activities of AB_SH in dry eye disease. Fucoidan also has a potent protective effect on dry eye disease. AB_SH and fucoidan may work synergistically to prevent hyperosmotic stress-induced ocular injury, even though we did not confirm detailed mechanisms.

*S. honeri* extract and fucoidan have potent anti-inflammatory and anti-apoptotic activities in various cells. *S. honeri* extract attenuated H_2_O_2_-induced apoptosis through free radical scavenging activity in C2C12 murine skeletal muscle cells [33]. *S. honeri* extract reduced neuroinflammation by inhibiting neuroinflammatory factors and NF-κB signaling in microglia cells [34]. Herath et al. reported that *S. honeri* extract suppressed particulate matter-induced cytotoxicity and inflammation by the inhibition of TLR2/4/7-MyD88-TRAF6-dependent signaling in the alveolar epithelial cells [35]. Fucoidan inhibited human neutrophil apoptosis and the production of pro-inflammatory cytokines [36]. Kizilay et al. showed that fucoidan prevented testicular tissue injury via the inhibition of apoptosis and ER stress in diabetic rats [37]. Song et al. reported that fucoidan attenuated ZnO nanoparticle-mediated autophagy and apoptosis through the regulation of the expression of proinflammatory cytokines in the rat brain [38].

Inflammation and apoptosis in ocular tissues can be induced during the development of dry eye disease [39,40,41,42]. Despite the fact that the precise mechanism by which apoptosis occurs in the dry eye has not yet been established, apoptosis in ocular tissues may play an important role in its pathogenesis [5,43]. Dry eye disease may be treated with therapeutic targets provided by the characterization of apoptotic pathways [42]. The FDA has approved the first treatment for dry eye disease, cyclosporine A. There was a decrease in apoptotic corneal epithelial cells after topical cyclosporine A treatment, decreased levels of proapoptotic factor p53, and increased levels of antiapoptotic factor Bcl-2 expression in eyes treated with topical cyclosporine A [44]. Unfortunately, anti-inflammatory activity of AB_SH was not evaluated in this animal model. However, recently, Lee et al. reported that AB_SH decreased the mRNA expression of IL-6 and IL-8 and the production of IL-6 and TNF-α in human retinal pigment epithelial cells. AB-SH also inhibited activation of the NF-kB/MAPK signaling pathway [13]. Accordingly, our results suggest that AB_SH administration may be beneficial for patients with dry eye disease through the prevention of apoptotic tissue damage.

In conclusion, *S. honeri* extract and fucoidan can protect corneal cells from hyperosmotic stress-induced apoptosis and ameliorate tear hyposecretion and apoptosis of the corneal epithelium and lacrimal gland. *S. honeri* extract and fucoidan may provide a beneficial option for patients with dry eye diseases.

## Figures and Tables

**Table 1:** Content of fucose in AB_SH.

Compound	Content (mg/g Dried Weight)
Fucose	277.1 ± 2.2

## Data Availability

The data presented in this study are available on request from the corresponding author.
